# Pick-Up of Organic Molecules by Mixed Ar Clusters: A Function of Gas Properties and Composition

**DOI:** 10.3390/molecules31030553

**Published:** 2026-02-05

**Authors:** Jernej Ekar, Oksana Plekan

**Affiliations:** 1 Elettra-Sincrotrone Trieste, AREA Science Park, 34149 Basovizza, Italy; oksana.plekan@elettra.eu; 2 Jožef Stefan Institute, Jamova Cesta 39, SI-1000 Ljubljana, Slovenia

**Keywords:** gas clusters, argon, hydrogen, doping, pick-up, mass spectrometry, pulsed gas beam

## Abstract

Clusters present an intriguing field of research, with their properties bridging the gap between an isolated atom/molecule and a bulk. They can act as a substrate for dopant molecules picked up on the fly and located on or inside the cluster. Our research on Ar clusters reveals that gas pressure and composition are crucial parameters determining the pickup probability for molecules such as adenine, uracil, glycine, and ascorbic acid. For pure Ar expansion, the most intense molecular signals are observed in the stagnation pressure range between 10 and 30 bar. Adding up to 33 mol% of He or O_2_ at fixed total pressure causes no change in the intensity of dopant and Ar oligomer signals. The addition of N_2_O or CO_2_ results in a significant intensity drop, with signals from the molecule and Ar oligomers disappearing above 3 mol% of N_2_O or CO_2_. The opposite effects are observed with the Ar-H_2_ mixture at a pressure of 25 bar. Optimal results are obtained for H_2_ concentrations between 40 and 50 mol% versus D_2_ concentrations between 20 and 35 mol%. Substitution of Ar with an Ar-H_2_ mixture causes signal intensities of dopants and Ar oligomers to increase by more than threefold.

## 1. Introduction

Clusters are intriguing nanoscale structures since their size and large surface-to-bulk ratio cause deviations from the bulk properties of materials with the same chemical composition [1,2]. Their properties can be considered intermediate between the gas and condensed phase [3]. Among the broad variety of clusters, gas-phase clusters are among the most straightforward to form since most gases only require expansion through an orifice into a vacuum [4]. Supersonic expansion generates clusters ranging from just a few to more than a billion atoms [5,6,7]. Their size can be easily manipulated via a few parameters, such as gas stagnation temperature and pressure, nozzle diameter and shape, and gas type, enhancing their applicability in nanoscience [8,9]. Growth of the cluster starts with the nucleation of single atoms and proceeds via the addition of individual atoms to the already formed cluster, as well as via the agglomeration of smaller clusters [10]. Lighter and less polarizable elements, such as H_2_, He, and Ne, experience weaker van der Waals interactions. Consequently, cooling the gas to below approximately 100 K is essential for the formation of H_2_, He, and Ne clusters [11,12,13,14,15,16]. While the majority of clusters are solid aggregates with an approximately spherical shape [5], H_2_ and He are known to form liquid clusters [17,18]. Liquid clusters become especially interesting in the scope of interactions with other chemical species [3]. This holds especially true for H_2_ and He clusters, which are known for their superfluidity [11,19]. As such, they offer a unique type of substrate for studying atoms and molecules with which they are doped, as well as interactions between them [20,21,22]. Ne clusters, also in combination with He, are studied from the point of isotopic enrichment important in astrophysics [23,24], while clusters of H_2_ isotopes are crucial in fusion studies [17].

Many different types of clusters exist, each with their unique properties. They range from mixed clusters composed of at least two different kinds of atoms or molecules [25,26,27], to doped clusters [28,29], to metal- and ionic-compound-based clusters [27,30,31]. Metal-based clusters are finding their way as electrode materials in energy storage materials [32]. In particular, doped clusters can also serve in the areas of catalysis and sensors [33,34]. Clusters containing free or bonded hydrogen isotopes serve in fusion experiments [15,35]. Water and ice clusters can help understand atmospheric chemistry [36,37] and also, in combination with other species, interstellar processes [38,39]. Clusters of versatile composition are becoming increasingly popular even in the areas of surface analysis, in combination with X-ray photoelectron spectroscopy (XPS) and secondary ion mass spectrometry (SIMS), where they serve for cleaning the surface and for depth profiling of sensitive organic and biological materials [40,41,42,43,44].

**Scheme 1:**
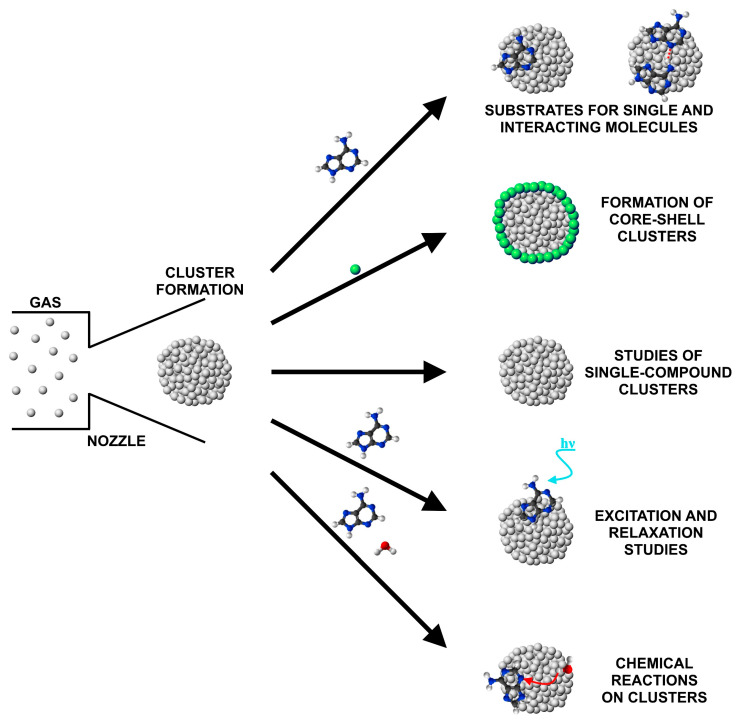
Some of the applications of gas clusters: substrates for single molecules, their dimers, and higher oligomers; formation and investigation of core–shell and uniform clusters; excitation and relaxation studies; and chemical reactions.

The compositional and structural diversity of clusters is central not only in the study of their intrinsic properties but also in their use as substrates for other atoms and molecules [3,18]. A cluster formed in a supersonic expansion and passing through a vapor can pick up atoms or molecules, whose type, along with the composition of the cluster and the mechanism of the pick-up process, all determine whether a given species will reside on the cluster’s surface or inside of it [22,45,46,47]. Binary clusters show, at least in some specific cases, segregation of the two constituents and formation of a compositionally different core and shell [48,49,50,51]. Numerous studies and experiments can be conducted after a compound binds to the cluster. The most straightforward approach is to measure the spectroscopic properties of individual atoms and molecules [52,53,54]. In the next stage, studies of atomic or molecular dimers, trimers, and higher oligomers can be performed [55,56,57], as well as research on excitation and relaxation mechanisms [58,59]. It is also possible to investigate reaction dynamics and kinetics of bound species and molecules colliding with clusters [60,61]. These can be simple reactions, such as deprotonation, or more complex reactions between different chemical species [62,63]. Clusters can even serve as a protective environment, preventing excessive fragmentation when probing organic molecules in the gas phase [64]. Different applications of clusters are graphically presented in Scheme 1.

This article focuses on the study of the pick-up properties of gas clusters, which determine the concentration of the dopant compound on the cluster. The pick-up probability can be defined as the probability of the atom or molecule being picked up by a passing cluster [65]. This probability is influenced by the column density and temperature inside the pick-up cell, the type of picked-up molecule, and cluster properties, including their composition, size, shape, temperature, and stability [65,66]. In the scope of our experiments, the pick-up probability is intended as the overall probability of the molecule being carried from its source to the detector via a gas-cluster beam. The primary gas used during our experiments was Ar, with pure Ar clusters being the only compositionally pure clusters that were formed. Mixed clusters were obtained with the addition of H_2_, He, O_2_, N_2_O, or CO_2_ to the Ar. Ar clusters were used as a basis since they are simple to form and consequently applied to many research field where doping of these clusters can pose breakthroughs [41,67,68,69]. Gas clusters were formed with a pulsed Even Lavie (EL) valve [70], and the pick-up of molecules occurred when clusters traveled through a heated pick-up cell.

Four different organic compounds (adenine (A), C_5_H_5_N_5_; uracil (U), C_4_H_4_N_2_O_2_; glycine (G), C_2_H_5_NO_2_; and ascorbic acid (AA), C_6_H_8_O_6_) were used as a source of molecular vapors. Different compounds were used to show that the studied properties of clusters are not compound-specific but rather have broad applicability. These specific compounds were used because of their biological significance and consequent importance in different research fields. High-resolution spectroscopic studies of cold, isolated biomolecules offer valuable starting points for understanding the potential energy surfaces (PESs) governing different decay channels. Moreover, it is crucial to determine how these PESs, especially key regions such as conical intersections, are modified by environmental interactions. As examples, adenine, uracil, and glycine were previously studied in their ground and excited states (pump–probe experiments) [71,72,73,74,75], proving they are of interest to even one of the most current state-of-the-art scientific communities with ongoing research on clusters. Molecular and Ar oligomer signals were quantified with a quadruple mass spectrometer (QMS) and read out by an oscilloscope. Pick-up probability was studied as a function of (1) the composition of gas mixtures and clusters, (2) the stagnation pressure of gases and their mixtures, and (3) the heating temperature of the organic compound. All three factors contribute to the molecular and Ar oligomer signal intensities, but the most significant effects were observed for variation in the gas composition. The highest pick-up efficiencies were achieved with clusters formed from an Ar-H_2_ mixture. Our findings can most probably be extrapolated to other compounds with different molecular structures and polarities. However, since a large fraction of organic materials have very low vapor pressures and easily decompose, a primary requirement is their sufficiently high vapor pressure, ensuring that sublimation can occur without inducing thermal decomposition.

## 2. Results

### 2.1. Effects of Ar Pressure

**Figure 1:**
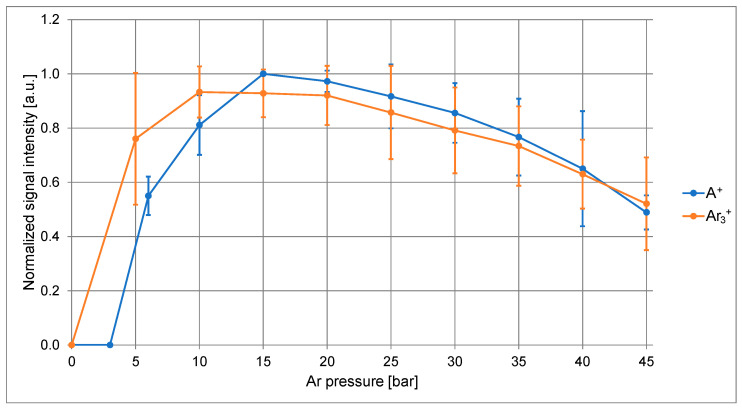
Normalized intensities of adenine (A^+^) and Ar_3_^+^ signals as a function of Ar pressure. Adenine powder was evaporated between 157 and 169 °C, and the pick-up cell was heated to between 137 and 157 °C during Ar oligomer measurements. The EL valve was kept at RT.

The change in intensity of molecular and Ar oligomer signals versus stagnation pressure was initially examined for pure Ar to determine the approximate working conditions for the mixed-gas experiments. Measurements of the organic compound listed above were repeated to obtain further insight. For adenine, three different signals were observed with the QMS: A^+^ (*m*/*z* = 135), [A + H]^+^ (*m*/*z* = 136), and A_2_^+^ (*m*/*z* = 270). Presence of dimers indicates molecular interactions on clusters and their simultaneous desorption after ionization, caused by interactions with the electrons in the QMS. Figure 1 presents the normalized A^+^ signal intensity as a function of Ar pressure, with the maximum between 15 and 20 bar of Ar. A^+^ signals measured during a given experiment were normalized to the A^+^ signal measured at the Ar pressure when the signal intensity was the highest. In this way, different experiments could be directly compared with each other. The same normalization approach applies to signals at other masses and all experiments. Since all three adenine signals show the same trend, with the only difference being the absolute intensity ratios of different signals, the other two signals ([A + H]^+^ and A_2_^+^) are omitted from Figure 1 for clarity. Furthermore, the A^+^ signal is only partly correlated to the firing of the EL valve: the associated pulsed signal rides on top of a relatively high background signal, present due to the effusive beam generated by the pickup cell. To account for this background, which stays approximately constant at a given cell temperature, the intensity of the A^+^ signal is defined as the difference between the intensity of the pulse peak and its baseline. Again, the same principle applies to all other signals with significant background intensity.

Ar oligomer signals were also examined, and the change in the Ar_3_^+^ (*m*/*z* = 120) normalized cluster intensity with the Ar pressure is also shown in Figure 1. Ar_2_^+^ (*m*/*z* = 80) and Ar_5_^+^ (*m*/*z* = 200) clusters were examined as well but are again omitted from the graph due to having the same pressure-dependent trend as Ar_3_^+^. Maxima for all three Ar oligomer signals can be observed between 10 and 20 bar of Ar. Pressure-dependent signal intensity scans were also performed for glycine and ascorbic acid, and the results do not deviate significantly from what was observed while using adenine. The maximum of the ascorbic acid fragment signal (C_4_H_4_O_4_^+^, *m*/*z* = 116) can also be observed between 10 and 20 bar of Ar, and the maximum of the glycine aminomethyl fragment signal (CH_4_N^+^, *m*/*z* = 30) can be observed at a slightly higher pressure, between 25 and 30 bar of Ar. The change in the normalized intensity of specific molecular fragments for glycine and ascorbic acid with Ar pressure is shown in Figure S1 in the Supplementary Information (SI) file. The Ar pressure-dependent measurements were also performed at different heating temperatures for adenine (157 and 169 °C) and glycine (125 and 142 °C). The EL valve was kept at room temperature (RT) during all the experiments examining both pressure- and gas-composition-dependent signal intensities. It was observed that heating the organic compound to different extents does not affect the position of the pressure-dependent signal maximum. In contrast, the absolute intensity of the molecular signal varies. The differences in the density of molecular vapors are the reason for the absolute intensity change. On the other hand, the signal intensity trend and the position of the pressure-dependent maximum are primarily a function of Ar cluster properties, whose formation is not affected by the heating temperature of a given compound. Only a molecular vapor density high enough to cause disintegration and extensive scattering of clusters could result in a change in the signal trend and in a shift in its maximum. The absence of such observations indicates that vapor densities are below this critical limit.

### 2.2. Effects of Ar Mixtures with H_2_, He, O_2_, N_2_O, and CO_2_

**Figure 2:**
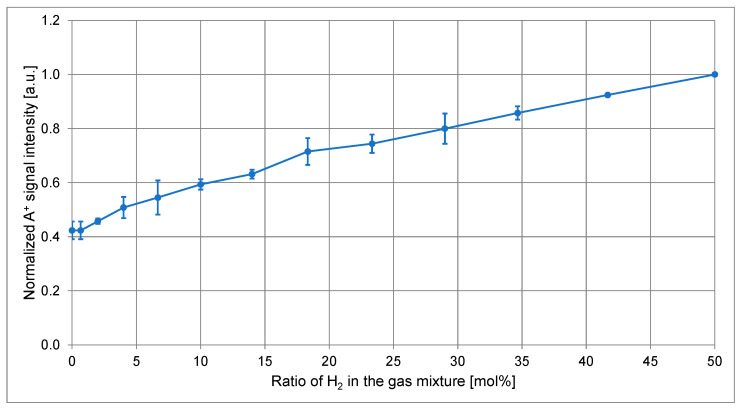
Normalized adenine (A^+^) signal intensity as a function of molar percentage of H_2_. Adenine powder was evaporated at 157 °C. Cluster source conditions: Ar-H_2_ mixture at 25 bar/RT.

Five additional gases were used to examine the pick-up properties of mixed Ar clusters: H_2_, He, O_2_, N_2_O, and CO_2_. The most significant differences were observed when Ar was mixed with H_2_ prior to gas expansion and formation of clusters. Such mixed Ar-H_2_ clusters significantly improved the pick-up efficiency for different organic molecules. The intensities of signals at the QMS (parent ion and fragments alike) for all four molecules examined, A, U, G, and AA, were the highest when gas clusters were formed from Ar-H_2_ mixtures with a molar percentage of H_2_ between approximately 30% and 50%. The intensities of these signals gradually dropped as the ratio of Ar was increased above 70 mol%. Figure 2 presents the normalized A^+^ signal intensity as a function of the molar percentage of H_2_ while heating adenine to 157 °C. The absolute pressure of the Ar-H_2_ mixture was 25 bar. Observation of the A^+^ signal shows an intensity increase caused by adding H_2_ at between 2- and 2.5-fold. The experiment with adenine was repeated at 169 °C, and normalized intensities of adenine signals (A^+^, [A + H]^+^, and A_2_^+^) are shown in Figure S2. In this case, the relative intensity increase in the A^+^ signal caused by the H_2_ addition exceeds 3-fold, indicating the vapor density as the limiting factor in the enhancement that can be achieved with the addition of H_2_.

**Figure 3:**
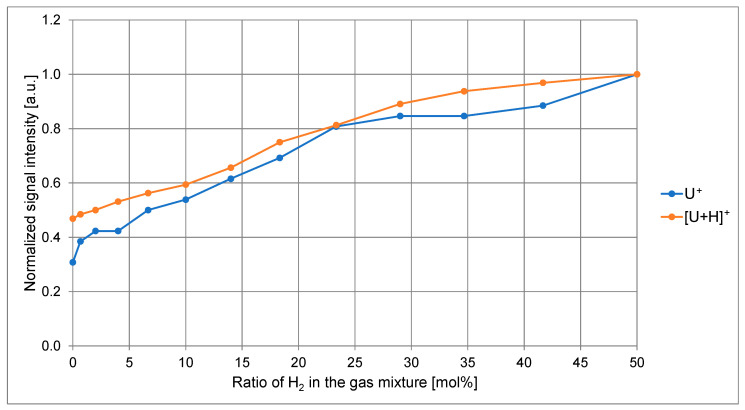
Normalized intensities of uracil (U^+^ and [U + H]^+^) signals as a function of molar percentage of H_2_. Uracil powder was evaporated at 152 °C. Cluster source conditions: Ar-H_2_ mixture at 25 bar/RT.

**Figure 4:**
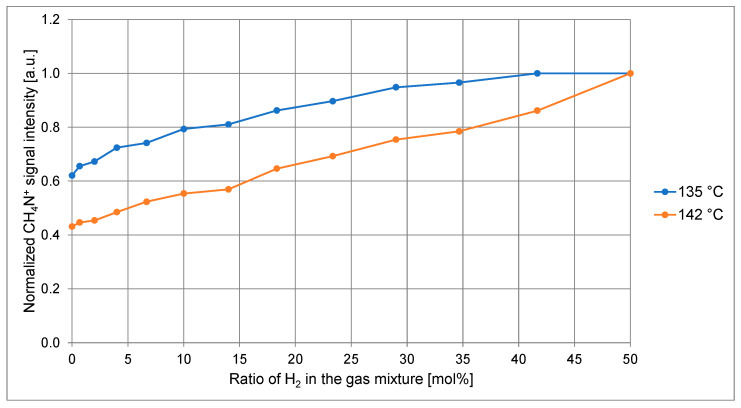
Normalized aminomethyl (glycine fragment CH_4_N^+^) signal intensity as a function of molar percentage of H_2_. Glycine powder was evaporated at 135 and 142 °C. Cluster source conditions: Ar-H_2_ mixture at 25 bar/RT.

A very similar temperature dependence was observed when uracil and glycine were analyzed. In the case of uracil, the molecular signal (U^+^) at *m*/*z* = 112 was again the most intense. In addition, the intensity of the [U + H]^+^ signal at *m*/*z* = 113 was measured. It was observed that heating uracil below 140 °C resulted in a vapor density in the pick-up cell that was too low, so no effects of H_2_ addition could be detected. When heating was increased to above 150 °C, the same effect was observed as with adenine, and uracil signal intensities were enhanced between 2- and 3-fold, as compared to pure Ar. Figure 3 shows normalized uracil (U^+^ and [U + H]^+^) signal intensities as a function of the molar percentage of H_2_ measured while heating the uracil to 152 °C. The pressure of the Ar-H_2_ mixture was 25 bar, which was also the case for the glycine measurements performed at 135 °C and 142 °C. The molecular signal of glycine at *m*/*z* = 75 was very weak, so the aminomethyl cation at *m*/*z* = 30 (CH_4_N^+^) was analyzed instead. Figure 4 shows the normalized CH_4_N^+^ signal intensity as a function of the molar percentage of H_2_ measured at both temperatures. The increase in the CH_4_N^+^ signal intensity caused by the addition of H_2_ can be determined to be approximately 1.5- and 2.5-fold when heating glycine to 135 and 142 °C, respectively.

**Figure 5:**
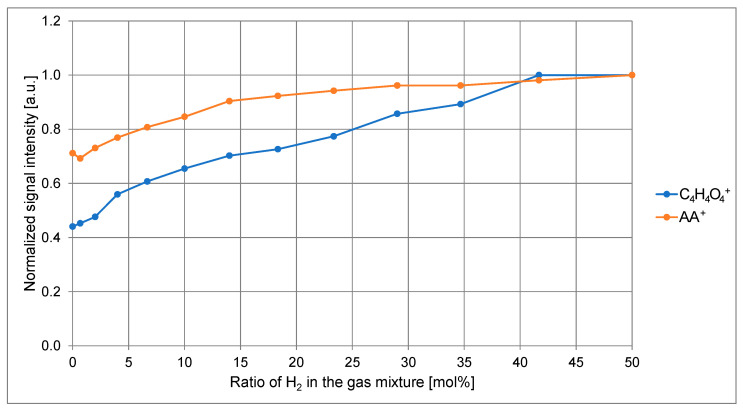
Normalized intensities of ascorbic acid (AA^+^ and fragment C_4_H_4_O_4_^+^) signals as a function of molar percentage of H_2_. Ascorbic acid powder was evaporated at 155 °C. Cluster source conditions: Ar-H_2_ mixture at 25 bar/RT.

Experiments with ascorbic acid were done at a single temperature of 155 °C. However, two different Ar-H_2_ mixture pressures were tested. Since the molecular signal of ascorbic acid at *m*/*z* = 176 (AA^+^) is relatively weak, the most intense fragment at *m*/*z* = 116 (C_4_H_4_O_4_^+^) was also measured. When the gas mixture pressure was 25 bar, the same H_2_ effect was observed as with other compounds. Figure 5 presents normalized intensities of signals from ascorbic acid (C_4_H_4_O_4_^+^ and AA^+^) as a function of the molar percentage of H_2_ measured at a stagnation pressure of 25 bar. The parent molecule (AA^+^) and the fragment (C_4_H_4_O_4_^+^) behave differently. The C_4_H_4_O_4_^+^ signal intensity increase caused by the addition of H_2_ was approximately 2-fold, while the increase in intensity of the AA^+^ signal was only 1.4-fold. An even larger difference was observed when measurements were done using an Ar-H_2_ mixture at a pressure of 15 bar. In this case, the C_4_H_4_O_4_^+^ signal intensity increase was 1.7-fold, and the most intense signal was measured at 34 mol% of H_2_. The change for the AA^+^ signal was even more pronounced since the lowest signal intensity was measured with the gas mixture containing 50 mol% of H_2_, and the highest signal intensity was measured with the mixture containing 20 mol% of H_2_. Figure S3 shows how changes in the H_2_ ratio affect the normalized intensities of signals of ascorbic acid measured at a gas mixture pressure of 15 bar. Such an effect of pressure indicates that the ratio Ar:H_2_ is not the only factor affecting the pick-up properties of gas clusters, and we should consider the overall gas and partial Ar pressures as well. The effects of these pressures can be extrapolated from the results obtained while testing pure Ar clusters at different gas pressures (Figure 1 and Figure S1). However, the relatively minor differences caused by the overall pressure change indicate that the gas composition affects the pick-up properties to a larger extent than its pressure. Differences between the parent ion signal (AA^+^) and the fragment (C_4_H_4_O_4_^+^) observed at a pressure of 15 bar also indicate changes in the fragmentation pattern caused by the addition of H_2_.

**Figure 6:**
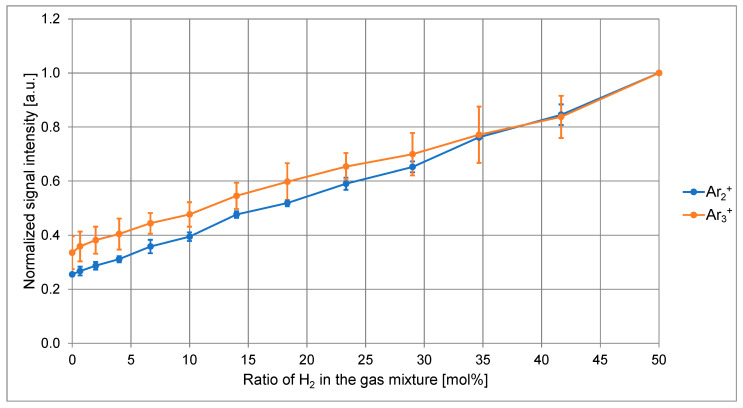
Normalized intensities of Ar_2_^+^ and Ar_3_^+^ signals as a function of molar percentage of H_2_. Cluster source conditions: Ar-H_2_ mixture at 25 bar/RT.

The dependence of signal intensities of Ar oligomers on the composition of the Ar-H_2_ mixture was very similar to what was measured for organic molecules. However, the intensity of Ar oligomers exhibited only minimal dependence on the heating temperature applied to organic compounds during the experiments, indicating that no significant disintegration or scattering of clusters occurred. Figure 6 shows the normalized Ar_2_^+^ and Ar_3_^+^ intensities as a function of the molar concentration of H_2_. The pressure of the Ar-H_2_ mixture was 25 bar, and changing it had no significant effect on the intensity trend of Ar oligomers. Their intensities were also measured with an Ar-H_2_ mixture at 15 bar, and the intensity enhancement caused by adding H_2_ remained approximately 3-fold. The signal maximum was observed in both cases for the mixture with a ratio of Ar and H_2_ of 1:1. Figure S4 shows how the normalized Ar_2_^+^ and Ar_3_^+^ intensities change with the changing Ar-H_2_ mixture composition at a pressure of 15 bar. Such observations indicate that the presence of H_2_ also affects the amount of Ar clusters, possibly at several stages (growth, ionization, and fragmentation).

**Figure 7:**
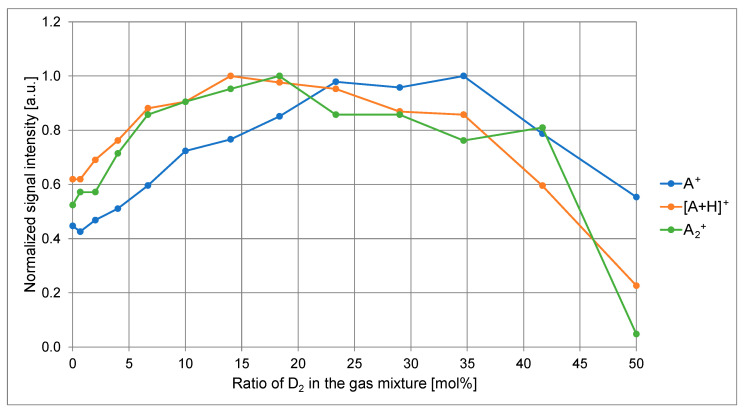
Normalized intensities of adenine (A^+^, [A + H]^+^, and A_2_^+^) signals as a function of molar percentage of D_2_. Adenine powder was evaporated at 162 °C. Cluster source conditions: Ar-D_2_ mixture at 25 bar/RT.

To help with a further evaluation of the underlying processes, especially concerning [A + H]^+^ and [U + H]^+^ cations, ^1^H_2_ was replaced with deuterium (D_2_) in an experiment with adenine. Figure 7 presents the normalized adenine (A^+^, [A + H]^+^, and A_2_^+^) signal intensities as a function of the molar percentage of D_2_. Measurements were performed at an adenine temperature of 162 °C and at a pressure of the Ar-D_2_ mixture of 25 bar. The signal at *m*/*z* = 137 was also analyzed, but insufficient mass resolution made it impossible to directly discriminate between [A + D]^+^ and [A + 2H]^+^. However, the *m*/*z* = 137 signal was present and had a relatively high intensity, also when using pure Ar without any D_2_ addition (approximately 3-times as high as when the ratio of Ar and D_2_ is 1:1), and its intensity trend was precisely the same as for the [A + H]^+^ signal. Consequently, it is possible to conclude that the [A + 2H]^+^ cation represents the majority contribution to this signal. Such an observation also indicates that H_2_ gas is not the primary source of hydrogen atoms for the formation of [A + H]^+^ and [A + 2H]^+^ cations. Furthermore, many differences were observed when comparing the addition of D_2_ to the addition of H_2_. The Ar-D_2_ mixture caused a shift in the signal maximum for A^+^ to a mixture with 35 mol% of D_2_ and for [A + H]^+^ and A_2_^+^ to a mixture with 14–18 mol% of D_2_. In the cases of [AD + H]^+^ and AD_2_^+^, the signal minimum was observed for the mixtures with the highest percentage of D_2_ (Figure 7). Therefore, this experiment’s results were similar to those obtained with ascorbic acid and the lower (15 bar) pressure of the Ar-H_2_ mixture. Considering the virtually identical chemical properties and very similar physical properties of H_2_ and D_2_, their difference in mass appears to be the major factor determining their somewhat different effects. The average molar mass of the gas is plainly affecting its forward speed [10], which was also observed in our experiments. When using an Ar-H_2_ mixture with ratios of Ar and H_2_ of 1:1 (molar mass 21 g mol^−1^), molecules and clusters arrived at the QMS in slightly more than 60% of the time needed for them to arrive when using pure Ar (molar mass 40 g mol^−1^). This translates to approximately a 1.6-fold increase in the speed of the clusters. Such observations are in relatively good agreement with the kinetic theory of gases, which states that the speed of a gas is inversely proportional to the square root of its molar mass [76].

Experiments with He, O_2_, N_2_O, and CO_2_ also indicate an effect of the molecular mass of the gas, but only to a limited degree and in combination with other properties. Addition of up to 30 mol% of He or 33 mol% of O_2_ caused no statistically significant change in the intensity of the adenine and Ar oligomer signals compared to their intensities measured in pure Ar at the same total pressure, which was 22 bar. This indicates that He and O_2_ have little effect on the pick-up properties of clusters. At the same time, a partial pressure of Ar between 15 and 22 bar is expected to cause no more than 10% of the change in the measured signal’s intensity, as shown in Figure 1 for the Ar pressure-dependent intensity trend of A^+^ and Ar_3_^+^ signals. This is in the statistical error range. One experiment was also performed with pure He as a carrier gas for adenine. Still, no signal originating from the adenine molecule was detected apart from the background, which is present even without any gas flow. Considering that the cluster source was kept at room temperature, such results are expected, since it is widely known that He requires extensive cooling to below approximately 100 K to form clusters [11,12,13,14]. The addition of N_2_O and CO_2_ to Ar had an effect, but it was the opposite of what was observed with H_2_. Adenine and Ar cluster signals only appeared after the ratios of N_2_O and CO_2_ in the gas mixtures with Ar fell below 2–3 mol%. Furthermore, any addition of N_2_O or CO_2_ in concentrations between 0.1 and 3 mol% resulted in signals having significantly lower intensities than in pure Ar.

## 3. Discussion

It is self-evident that adding H_2_ to Ar while keeping the overall pressure constant reduces the partial Ar pressure. As observed from Figure 1 and Figure S1, changes in Ar pressure result in either improvement or deterioration in the pick-up properties of clusters. However, when considering changes caused by the addition of H_2_, the range spanned by the partial Ar pressure (from 12.5 bar to 25 bar and from 7.5 bar to 15 bar) is relatively small. Differences in pick-up properties caused by this change alone should not exceed a factor of 1.3, as deduced from the pressure dependence of A^+^ and Ar_3_^+^ signals in Figure 1. On the other hand, as observed from Figure 2, Figure 3, Figure 4, Figure 5 and Figure 6, the addition of H_2_ improves pick-up probability by more than 3-fold. Changes in the partial Ar pressure caused by the H_2_ addition have an even lesser effect on Ar oligomer intensities. As observed from Figure 6 and Figure S4, lowering the total pressure from 25 to 15 bar does not change the intensity trend of Ar_2_^+^ and Ar_3_^+^ at all, with their intensity maxima always found for mixtures with 50 mol% of H_2_. Such observations lead to the conclusion that the changed partial Ar pressure caused by the H_2_ addition is responsible only for the minor signal intensity changes. The discussion evaluating the changes in the chemical and physical properties of mixed clusters as the primary effect follows.

Changing the composition of the Ar-H_2_ gas mixture and/or the partial Ar pressure results in a different Ar cluster size distribution. The size distribution obtained during our study cannot be determined experimentally, since the QMS used for the analysis detects ions only up to *m*/*z* = 1000. However, knowing the parameters of the EL valve and the operational conditions, the size of Ar clusters can be calculated with the help of Equations (1) and (2) [8,9].

(1) $$\langle N\rangle =2.94\times 10^{-6}\left(\frac{k^{2.35}d_{eq}^{2}P_{0}^{2.35}}{T_{0}^{5.38}}\right)$$

(2) $$d_{eq}=\frac{0.74d}{\tan\alpha }$$

Here, $\langle N\rangle$ represents the average cluster size, and *k* represents a constant with the value of 1650 in the case of Ar [9]. *d_eq_* is the equivalent nozzle diameter expressed in µm, *d* is the conical nozzle’s diameter, and *α* is half of the cone angle. In the case of the EL valve used during the experiments, the value of *d* equals 150 µm, and half of the cone angle is 20° [70,77]. *T*_0_ is the stagnation temperature of the gas in K. Since operation of the valve causes slight Joule heating, its temperature was just above 300 K. The gases were fed into the valve at room temperature, and the difference can be neglected, so the approximation of *T*_0_ = 300 K is appropriate. *P*_0_ represents the stagnation gas pressure in mbar. The pick-up of organic molecules as a function of size of pure Ar clusters can be assessed by relating Figure 1 and Figure S1 with the scaling law described via Equations (1) and (2). The presence of a signal intensity maximum between 10 and 30 bar shows a certain optimal stagnation pressure range. Equation (1) indicates that the average cluster size increases with increasing pressure by a power of 2.35. Simultaneously, the number (molar) density of gas increases with the first power of pressure, as described by Equation (3), where *ρ_N_* represents the number density and *k_B_* represents the Boltzmann constant. Comparison of Equations (1) and (3) shows that the average cluster size increases faster than the number of gas particles, resulting in a decrease in the number of clusters with increasing stagnation pressure. Therefore, the signal maxima observed in Figure 1 and Figure S1 indicate the optimal number/size of clusters ratio rather than optimal cluster size when considering molecular pick-up.

(3) $$\rho_{N}=\frac{P}{k_{B}T}$$

The behavior of gas mixtures is, from the point of the scaling law, more complicated. If both gases in the mixture condense and form clusters under these conditions, then their scaling law constants *k* should be considered together with the overall pressure [15,78]. In such cases, clusters enriched in a heavier gas are predominant [78]. However, H_2_ by itself, similarly to He, forms clusters only under cryogenic conditions [15]. The approximation of considering *P*_0_ only as a partial Ar pressure in the overall range between 7.5 and 25 bar was consequently adopted. It needs to be emphasized that this is only an approximation, potentially introducing an error, since the use of the scaling law for gas mixtures is problematic. In particular, even if one could assume that the size distribution (Equations (1) and (2)) remains the same when *P*_0_ is the partial pressure of Ar, the fraction of condensed Ar can be expected to be larger for the mixture, since gases with low condensation temperatures, such as H_2_, significantly contribute to the evaporative cooling. Of course, the overall pick-up efficiency is influenced by both the size and number of clusters. Nevertheless, a similar, albeit not the same, simplification that disregards He pressure in the mixture has already been used in the past [9]. A scattering-based study also indicates that true Ar-H_2_ mixed clusters are formed in only minimal amounts or even not at all if the concentration of H_2_ in the Ar-H_2_ gas mixture is below 40% [15]. Considering the listed conditions, the approximate average Ar cluster size $\langle N\rangle$ ranges from 600 atoms at 7.5 bar to 10,000 atoms at 25 bar. While pure H_2_ does not form clusters at room temperature, it was shown that mixed Ar-H_2_ clusters are formed under such conditions, at least if the H_2_ concentration exceeds 40%. The Ar-H_2_ cluster size reaches a maximum at an H_2_ ratio of 60%, but mixed Ar-H_2_ clusters remain smaller than the pure Ar clusters formed at the same overall pressure [15]. The size of the mixed clusters formed at the partial Ar pressure of 7.5 bar (overall pressure of 15 bar) is consequently larger than 600 atoms, but there is no quantitative approach towards determining the exact size. Nevertheless, a reduction in the average gas cluster size caused by the H_2_ addition is expected, while there is also a possible increase in the number of formed clusters.

In addition to their size and number, the cluster shape should be considered. Larger clusters are formed by adding new atoms to the existing smaller cluster and by the agglomeration of such smaller clusters [10]. Colliding clusters can fully coalesce, forming a new spherical cluster [5], but this is not the only option. Newly formed larger clusters could potentially take irregular shapes of agglomerated smaller clusters, and the structure and interactions between these clusters can influence the evolution of the coalescence, as well as the pick-up properties. For Ar-N_2_ mixtures, clusters are formed with N_2_ segregated in the outer layers [48]. This is in line with a general theory of core–shell separation occurring in the case of mixed clusters formed from constituents with substantially different condensation temperatures. In such cases, the gas with the higher condensation temperature forms the core, and the gas with the lower condensation temperature forms the shell, as their mixing by diffusion is highly limited due to the low temperature of the core [9,78]. If a similar separation, which has not yet been proven, indeed happens in the case of an Ar-H_2_ mixture, complete coalescence of small clusters may be limited by the interactions caused by the compositionally different cluster core and shell. Such an irregularly shaped cluster could also explain the increased pick-up probability observed for mixed Ar-H_2_ clusters. Spherical clusters primarily have a convex surface, which is not ideal for the molecule or atom to stick to. On the other hand, irregular shapes provide concave areas, such as cavities and valleys, in which molecules and atoms can get trapped. Reduction in the average cluster size and/or formation of irregularly shaped agglomerates also increases the surface-to-volume ratio, providing more potential binding sites for the organic molecules. The heating temperature-dependent observations, most clearly visible for glycine in Figure 4, also align with cluster size and shape explanations. The signal intensity enhancement can only be observed above a compound-specific lowest heating temperature, followed by an increase in this effect with increasing temperature. At a vapor density that is too low, it seems that Ar clusters can pick up the majority of molecules by themselves, so the H_2_-induced improved pick-up probability cannot be observed. When the concentration of molecules becomes high enough, spherical clusters might no longer provide enough support to carry all these molecules, while the agglomerates do. However, at the current research stage, this is only a hypothesis requiring further evidence. H_2_-addition-induced improvements at higher vapor densities can be explained by the molecule–molecule and molecule–cluster interactions as well, with the latter being expressed as a sticking probability [3,79].

Besides influencing the chances of a successful collision between the cluster and the molecule, changes in the cluster composition can also affect where on/in the cluster the molecule will reside. Mixed clusters with compositionally different cores and shells were even shown to possibly have solid cores and liquid shells [45]. If separation is also present in the mixed Ar-H_2_ clusters, their significantly different melting and boiling points could lead to a similar phenomenon, which would cause a profound difference in comparison to the solid pure Ar clusters [5]. H_2_ clusters are, similarly to He clusters, fluids [60], further supporting such an explanation. Different boiling points of Ar and H_2_ can also result in different final temperatures of pure Ar and mixed Ar-H_2_ clusters, as determined by evaporative cooling [80]. The additional cooling of Ar induced by the H_2_ evaporation can result in more stable clusters and different, probably enhanced, sticking and pick-up probability. However, heating of the H_2_ during its expansion at room temperature as a consequence of the Joule–Thompson effect should be considered as well [81,82]. Even without changes in the aggregation state of the cluster, it was shown that factors such as the size of the cluster and the species being picked up already influence the location of their residence [3,53,83,84,85]. The critical factor influencing this process is the solvation energy compared to the surface energy of different species [85,86]. Formation of dimers and fragmentation observed in Figure S2 and Figure S3 indicate that the addition of H_2_ might indeed affect the exact position, depth, and cluster environment surrounding the molecule. The signal maxima for the molecular peak of the ascorbic acid (AA^+^), adenine dimer (A_2_^+^), and [A + H]^+^ appear at the lower H_2_ concentration in the gas mixture compared to the ascorbic acid fragment (C_4_H_4_O_4_^+^) and adenine (A^+^). Such observations indicate that increasing H_2_ concentration isolates picked-up molecules and/or causes them to be more selectively surface-bound and exposed to the electron bombardment in the QMS. The latter will increase ionization yield but also enhance the molecular fragmentation rate.

Similar results, indicating the same mechanism, were observed for the Ar-D_2_ mixture, where intensity maxima for [A + H]^+^ and A_2_^+^ signals appear at a lower D_2_ concentration than in the case of the A^+^ signal (Figure 7). Furthermore, the high intensity of the [A + H]^+^ signal and low intensity of the [A + D]^+^ signal suggest weak interactions between picked-up molecules and H_2_ or D_2_. Protons, required for the formation of the [A + H]^+^ ion, consequently originate from other species, and the correlation between the [A + H]^+^ and A_2_^+^ signals indicates possible proton transfer between individual molecules. Intermolecular interaction in the form of proton transfer or bonds suits this explanation because intensity maxima of [A + H]^+^ and A_2_^+^ signals are observed at approximately the same H_2_ or D_2_ concentration, and the proximity of two molecules is required for both proton transfer and dimer formation. When comparing Figure 2, Figure 7 and Figure S2, an overall shift in the signal intensity maxima from the higher H_2_ concentrations to the lower D_2_ concentrations can also be observed. Since H_2_ and D_2_ have virtually identical chemical properties and very similar physical properties, the difference in their masses has to play at least a partial role in determining the optimal conditions for the molecule pick-up. The mass change affects the speed of the clusters, as most clearly observed when comparing pure Ar and an Ar-H_2_ mixture. The difference in mass, and consequently speed, between Ar-H_2_ and Ar-D_2_ clusters is much smaller but still present. Changes in the speed of the clusters also affect the pick-up cross-section and therefore the pick-up probability, with increasing speed reducing the pick-up cross-section [3,5]. Such an effect is opposite to the results observed during our experiments, where the fastest clusters picked up the most molecules. Furthermore, faster clusters spend less time in the ionization region of the QMS, lowering the ionization probability, which is already below 1‰ for the electron ionization [87]. This implies that the ionization probability will scale approximately with the inverse speed of the clusters. H_2_ addition causes up to a 1.6-fold increase in the speed of the clusters, resulting in a decrease in ionization probability and signal intensities for that same factor. However, these contributions do not disprove the correlation between the pick-up cross-section and the speed of the clusters but only indicate that other effects, matching the H_2_-induced increase in the molecule pick-up, contribute more significantly than the change in the cluster speed. Considering ionization probability alone, this also means that signals measured in Ar-H_2_ mixtures should have up to almost 40% greater signal intensities if not for the ionization probability change. Changes in pick-up cross-section and ionization probability also indicate that the mass change, as observed for the H_2_-D_2_ comparison, has to influence the pick-up probability via different means of action. Still, its exact evaluation and specific determination are not possible with the results of these experiments. If the changed speed of the clusters was the only factor contributing to the differences between H_2_ and D_2_, then the opposite effect from what was observed should be detected.

Since organic molecules are bound to the cluster, their ionization in the QMS cannot be considered as an ionization of an isolated molecule. A change in the cluster composition consequently changes the substrate where the ionization occurs. A change in the substrate changes the electronic properties, including ionization potentials, and the composition and structure of clusters affect the possibility of charge transfer [83,88]. These inherently influence the ionization probability, which can be another factor contributing to the overall signal intensity differences observed during the experiments. However, the fact that a high enough concentration of organic molecules in the vapors is required for the H_2_ effect to be observed indicates that a changed ionization yield is probably not the main contributing factor. If it were, an increase in the molecular signal intensity would be present regardless of the heating temperature. The most important effects are probably connected with the pick-up process. Still, all of the discussed factors almost surely influence an increased pick-up probability achieved with the H_2_ addition to Ar, each contributing in a different, case-specific amount. However, verification and quantification of their individual contributions would require extensive molecular modeling using simulations. As such, this is out of the scope of this work but may be studied and evaluated in the future. This research can also provide the basis for future experiments with synchrotrons and free-electron lasers, with both of them available at the Elettra facility, where the present study was conducted. Photon-based experiments can be performed on pure and mixed clusters, as well as on molecules picked up by the clusters.

## 4. Materials and Methods

### 4.1. Materials

All the organic compounds used for the pick-up experiments were purchased commercially and used without further treatment. Adenine and glycine were obtained from Alfa Aesar (Ward Hill, MA, USA), and uracil and ascorbic acid were obtained from Sigma-Aldrich (Burlington, MA, USA). Gases used during the experiments (Ar, He, O_2_, H_2_, N_2_O, and CO_2_) were of analytical purity and commercially purchased. Adenine was heated to between 157 and 169 °C, uracil to 152 °C, glycine to between 125 and 142 °C, and ascorbic acid to 155 °C. These temperatures were determined via three factors. Firstly, they needed to be high enough to vaporize the compounds as a primary requirement for the pick-up by the clusters. Secondly, they were kept low enough to maintain a low-intensity effusive beam, which led to an increase in the background. The ratio of cluster-specific signal to background decreases at a very high rate of vaporization, leading to larger measurement errors. Lastly, too high sample-specific temperatures also cause decomposition of such organic compounds, so care was taken not to exceed these limits.

### 4.2. Gas Cluster Formation

Clusters were generated with an Even Lavie (EL) pulsed valve [70]. The valve had a conical expansion nozzle with a 150 µm wide opening (diameter). The angle of the nozzle was 40°. Pulse length (valve opening time) was set to 35 µs for all measurements. The gas cluster beam was further collimated with a conical skimmer (Beam Dynamics model 76.2, orifice 4 mm in diameter) placed approximately 12 cm after the valve. A heated pick-up cell containing the target organic compound was located approximately 15 cm after the skimmer’s orifice. The cluster beam propagated through the cell via entrance and exit holes with a diameter of 2.5 mm. The path length of the beam inside the pick-up cell was 1 cm. Approximately 5 cm after the pick-up cell were two piezoelectrically operated vertical slits (PZS 3, Piezosystem Jena, Jena, Germany) spaced 1.5 mm apart. The last beam-defining aperture with a diameter of 5 mm was at the entrance into the QMS chamber. The distance between the pick-up cell and the detection region inside the QMS chamber was slightly less than 1 m. Therefore, the distance between the front plate of the EL valve and the detection region was approximately 125 cm.

The gas from the EL valve was expanded into the chamber at a pressure between high 10^−6^ mbar and low 10^−5^ mbar, depending on the gas stagnation pressure. The pressure in the detection region was in the range between high 10^−9^ mbar and low 10^−8^ mbar due to differential pumping and a series of small apertures.

Gas mixtures were prepared in a cylinder with a volume of 1 L. The cylinder was initially filled with He, O_2_, H_2_, N_2_O, or CO_2_ at a pressure corresponding to the highest concentration of these gases in mixtures with Ar. Ar was added in the second stage in an amount providing both the desired concentration and overall stagnation pressure. The mixing cylinder was then isolated, and all the tubing was evacuated, ensuring residual Ar did not alter the composition of the injected gas in the tubes. The stagnation pressure was measured after refilling the tubing and valve with the gas mixture from the cylinder. The intensity of the molecular signal from the parent ion of the fragment was observed, and measurement was initiated only after this signal reached a steady-state region. This delay was introduced to compensate for any remains of unmixed gases. A similar mixing approach has already been proven to be effective [15]. Additional Ar was added after measurements at each He, O_2_, H_2_, N_2_O, or CO_2_ concentration to reach a new concentration point.

### 4.3. Signal Detection

Molecules and clusters were detected and quantitatively measured with a quadrupole mass spectrometer (MAX1000, Extrel, Pittsburgh, PA, USA). QMS extraction was oriented perpendicularly to the gas beam trajectory, while the potential of the extractor lenses was kept at 16 V (negative polarity). The electron ionization source of the QMS was set to 70 eV kinetic energy. *m*/*z* ranges applied for measurements of signal intensities were 0.1 mass unit wide (e.g., between 137.1 and 137.2). The specific range for each ion was determined manually by scanning different ranges and selecting the one that provided the highest signal intensity. This mass range was kept the same for all measurements of the selected ion. Mass calibration of the QMS was kept constant as well. Multiplier voltage was kept between 1500 V and 2000 V, determined individually for each ion to prevent saturation, and it was kept constant through all the measurements of the selected ion. QMS measurements were read out with a 1 GHz oscilloscope (WaveRunner 610Zi, Teledyne LeCroy, Thousand Oaks, CA, USA). Signal intensities for all the measurements were determined as peak heights due to the technical limitations of the oscilloscope. Peak area can only be measured by zooming in on the peak until it covers the whole oscilloscope screen. This approach is very imprecise due to the large zooming steps and extreme widening of the peak, preventing precise determination of its boundaries. Furthermore, subtraction of the background, caused by the effusive beam, is possible only when measuring the peak height and not when measuring its area. As such, measurements of the peak area would provide significantly underestimated values of the absolute intensity changes and prove to be imprecise and inaccurate. On the other hand, peak heights were slightly affected by a narrowing of the peaks observed in Ar/H_2_ mixtures, resulting in only a slight overestimate of the integrated signal. To increase data reliability, peak heights were sometimes still compared with peak areas. Such a comparison shows the same signal intensity trend and indicates that overestimation, caused by narrowing of the peaks, should not exceed 30%.

## 5. Conclusions

Pick-up properties of pure and mixed Ar clusters were studied using different organic compounds—adenine, uracil, glycine, and ascorbic acid. Effects of gas pressure, cluster composition, and temperature of the compound heating were examined. There was an Ar pressure range between 10 and 30 bar with an optimal pick-up efficiency and an ideal compound-specific heating temperature range. Below the minimal temperature, there was essentially no vaporized compound. In contrast, above the maximal temperature, the effusive beam of organic molecules caused such an intense background that no cluster pick-up effects could be observed. Gases used in the mixtures with Ar were He, O_2_, N_2_O, CO_2_, and H_2_/D_2_. Additions of up to 33 mol% of He or O_2_ caused no change in the intensity of the signals of the analyzed compounds measured with the QMS, so there was no change in the pick-up efficiency. Adding N_2_O or CO_2_ to Ar significantly reduced cluster formation. Only mixtures with less than 3 mol% of N_2_O or CO_2_ were able to pick up organic molecules and carry them to the QMS. Still, even mixtures with 0.1 to 3 mol% of N_2_O or CO_2_ caused molecular signal intensities to drop drastically compared to pure Ar clusters. The addition of H_2_ or D_2_ had an opposite effect, increasing the intensities of molecular and small Ar cluster signals even more than 3-fold. The highest intensities of these signals were observed for the mixtures with between 40 and 50 mol% of H_2_ when working under an overall gas pressure of 25 bar. A mixture with 50 mol% of H_2_ was also the most concentrated tested. Lowering the pressure to 15 bar caused a shift in the maxima of molecular ions and their fragments to between 20 and 35 mol% of H_2_. Replacing H_2_ with D_2_ had a similar effect, causing the molecular signal maxima to shift to between 15 and 35 mol% of D_2_ even under a pressure of 25 bar. Heating of the organic compound also had an effect, as high enough temperatures were required to observe the signal intensity enhancement caused by the addition of H_2_. Intensities of the molecular signals remained unchanged if the compound-specific temperature was too low. Numerous factors can explain these observations, each contributing in its specific way and proportion. The most important ones are:

- Size and shape of the gas clusters;
- Their potential core–shell compositional difference;
- Stagnation temperature and stability of clusters, combined with their aggregate state;
- Cluster speed and pick-up cross-section;
- Molecular mass of gases added to Ar, together with their physical and chemical properties;
- Localization of picked-up molecules on or inside the cluster;
- Changes in sticking and ionization probability;
- Partial Ar pressure and temperature of the organic compound heating.

## Data Availability

Signal intensities were read from the oscilloscope monitor, so they are only listed in an Excel sheet. The Excel file with signals presented and evaluated in the paper is available online, along with the Supplementary Information file. Conditions such as temperature and pressure are also listed. The authors of the paper should be contacted if anything in the Excel file is unclear. A few other signals were also measured, but since they are currently not systematized and organized, as they were not used to interpret the results, the authors of the paper should be contacted if anyone is interested in these signals as well.

## Supplementary Materials

The following supporting information can be downloaded at: https://www.mdpi.com/article/10.3390/molecules31030553/s1, Figure S1. Normalized intensities of ascorbic acid fragment (C_4_H_4_O_4_^+^) and aminomethyl (glycine fragment CH_4_N^+^) signals as a function of Ar pressure. Ascorbic acid powder was evaporated at 155 °C and glycine powder at 125 and 142 °C. The EL valve was kept at RT; Figure S2. Normalized intensities of adenine signals (A^+^, [A + H]^+^, and A_2_^+^) as a function of molar percentage of H_2_. Adenine powder was evaporated at 169 °C. Cluster source conditions: Ar-H_2_ mixture at 25 bar/RT; Figure S3. Normalized intensities of ascorbic acid (AA^+^ and fragment C_4_H_4_O_4_^+^) signals as a function of molar percentage of H_2_. Ascorbic acid powder was evaporated at 155 °C. Cluster source conditions: Ar-H_2_ mixture at 15 bar/RT; Figure S4. Normalized intensities of Ar_2_^+^ and Ar_3_^+^ signals as a function of molar percentage of H_2_. Cluster source conditions: Ar-H_2_ mixture at 15 bar/RT.
