# Supplementary material for: Pick-Up of Organic Molecules by Mixed Ar Clusters: A Function of Gas Properties and Composition
Source: Molecules. 2026 Feb 5;31(3):553. doi: 10.3390/molecules31030553 (PMC12899421; doi:10.3390/molecules31030553)
Supplement: Supplementary file 1 [file molecules-31-00553-s001.zip › Supplementary Information.pdf]

Supplementary Materials

Pick up of Organic Molecules by Mixed Ar Clusters: A Function of Gas Properties and Composition

Jernej Ekar [1,2,*] and Oksana Plekan [1]

[1] Elettra-Sincrotrone Trieste, AREA Science Park, 34149 Basovizza, Trieste, Italy; jernej.ekar@elettra.eu (J.E.); oksana.plekan@elettra.eu (O.P.)

[2] Jožef Stefan Institute, Jamova cesta 39, SI-1000 Ljubljana, Slovenia; jernej.ekar@ijs.si (J.E.)

* Correspondence: jernej.ekar@ijs.si (J.E.)

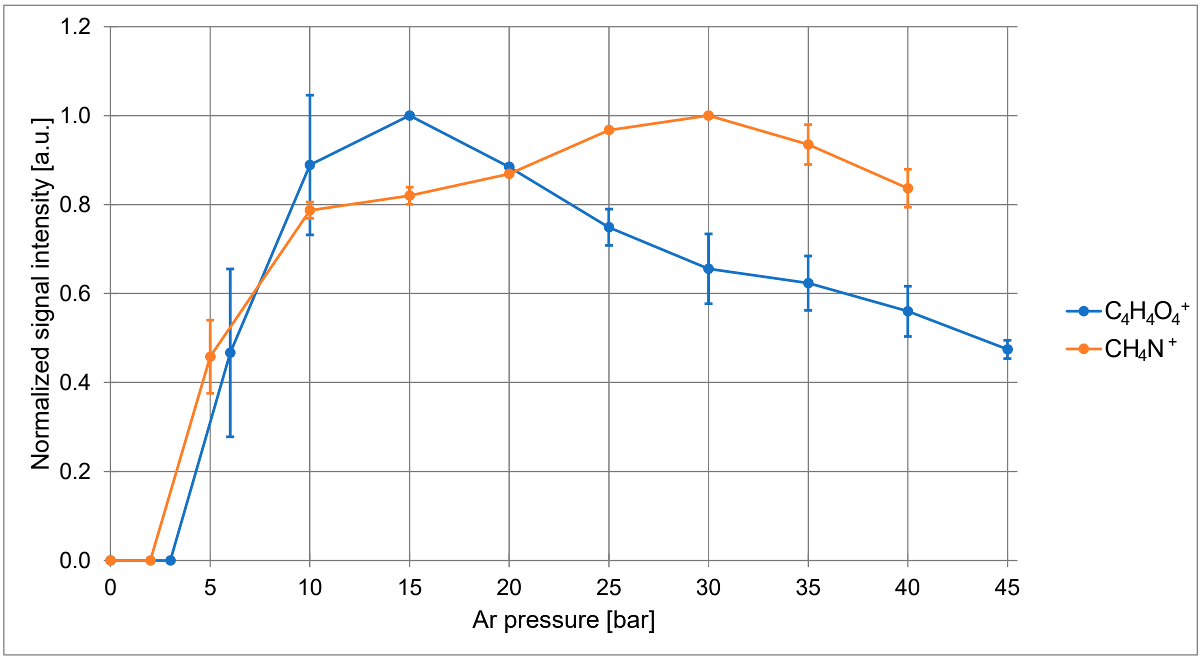

Figure S1. Normalized intensities of ascorbic acid fragment ($C_4H_4O_4^+$) and aminomethyl (glycine fragment CH_4N^+) signals as a function of Ar pressure. Ascorbic acid powder was evaporated at 155 °C, and glycine powder at 125 and 142 °C. The EL valve was kept at RT.

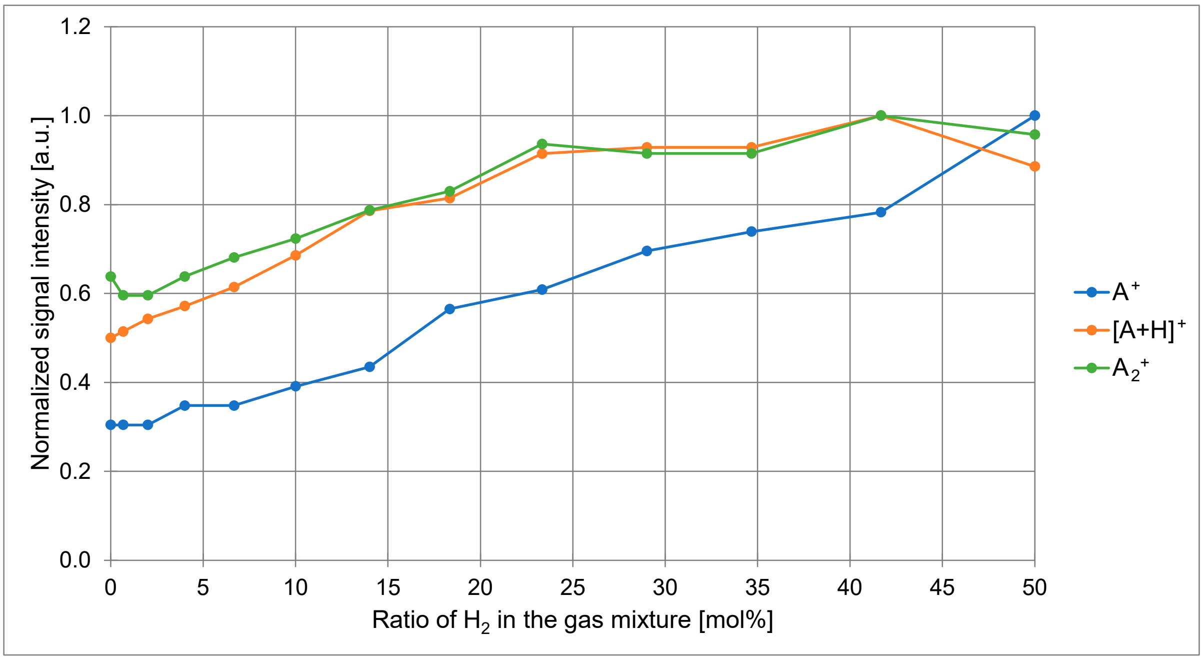

Figure S2. Normalized intensities of adenine signals (A^+, $[A+H]^+$, and A_2^+) as a function of molar percentage of H_2. Adenine powder was evaporated at 169 °C. Cluster source conditions: Ar-H_2 mixture at 25 bar / RT.

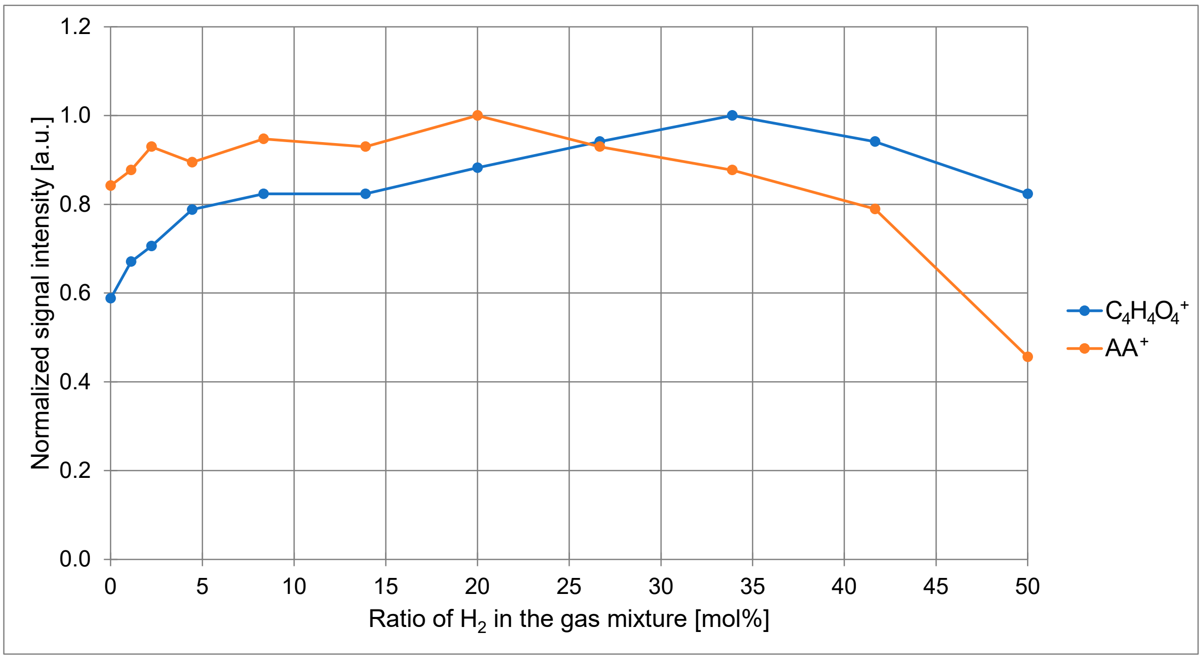

Figure S3. Normalized intensities of ascorbic acid (AA^+ and fragment $C_4H_4O_4^+$) signals as a function of molar percentage of H_2. Ascorbic acid powder was evaporated at 155 °C. Cluster source conditions: Ar-H_2 mixture at 15 bar / RT.

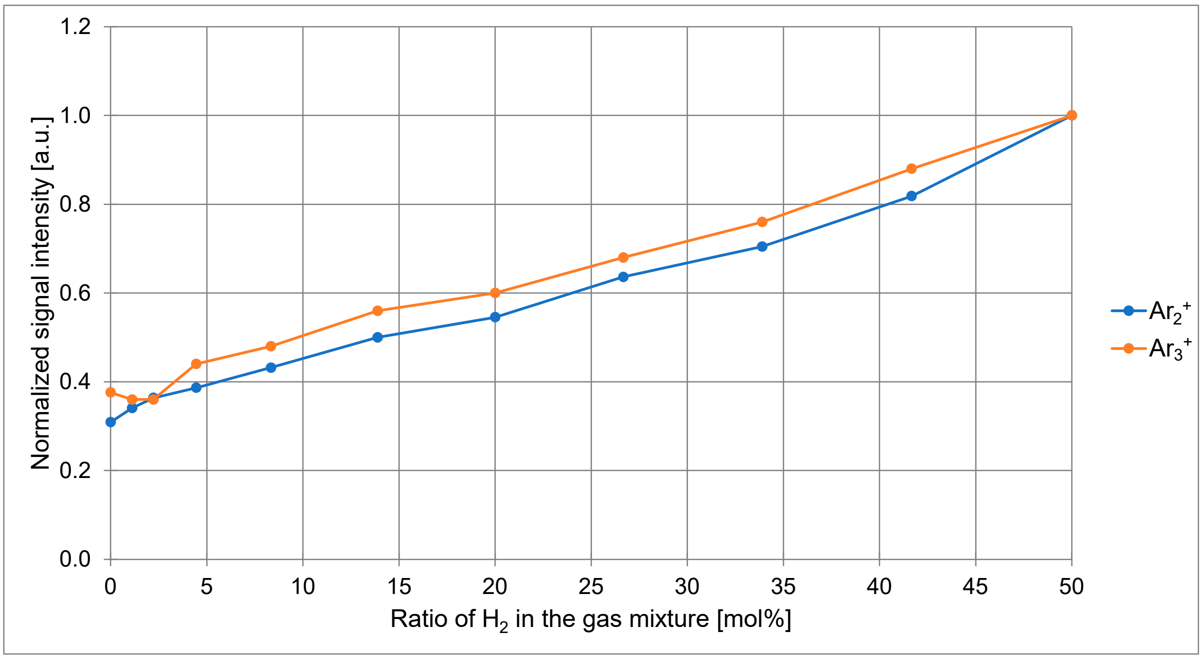

Figure S4. Normalized intensities of Ar_2^+ and Ar_3^+ signals as a function of molar percentage of H_2. Cluster source conditions: Ar-H_2 mixture at 15 bar / RT.
